# Activation of the Host Immune Response in *Hyphantria cunea* (Drury) (Lepidoptera: Noctuidae) Induced by *Serratia marcescens* Bizio

**DOI:** 10.3390/insects12110983

**Published:** 2021-10-30

**Authors:** Zhiqiang Wang, Kai Feng, Fang Tang, Meng Xu

**Affiliations:** 1 Co-Innovation Center for Sustainable Forestry in Southern China, Nanjing Forestry University, Nanjing 210037, China; WZQFAFU@163.com (Z.W.); fengkai@njfu.edu.cn (K.F.); xum@njfu.edu.cn (M.X.); 2 College of Forestry, Nanjing Forestry University, Nanjing 210037, China

**Keywords:** *Hyphantria cunea* (Drury), *Serratia marcescens* Bizio, insect–bacteria interaction, antimicrobial peptides, melanization, cellular immunity

## Abstract

**Simple Summary:**

*Hyphantria cunea* (Drury) is a quarantine pest, due to its extensive host, leading to serious economic losses in the agricultural and forestry industries. To control this pest, it is increasingly important to use microbial pesticides because they are biologically active and ecologically safe. *Serratia marcescens* Bizio (SM1) is a potential biocontrol bacterium. Although SM1 has a pathogenic role in *H. cunea*, *H. cunea* self-defense reduces the pathogenic effect of SM1. In this study, immune-related differentially expressed genes (DEGs) in *H. cunea* were first identified after SM1 infection, and the immune regulation mode of *H. cunea* in response to SM1, including antimicrobial peptide synthesis pathways, melanization and cellular immunity, was revealed. According to the analysis, the immune system of *H. cunea* was induced by SM1. In summary, our study demonstrates how the immune systems of the *H. cunea* work to resist the infection of SM1, which provides the theoretical basis for researching more efficient microbial pesticides for *H. cunea*.

**Abstract:**

Host–pathogen interactions are essential to our understanding of biological pesticides. *Hyphantria cunea* (Drury) is an important forest pest worldwide. The immune mechanism of the interaction between *H. cunea* and *Serratia marcescens* Bizio (SM1) is unclear. First, transcriptome sequencing and quantitative real-time PCR (qRT-PCR) analysis described the *H. cunea* immune response to SM1. A total of 234 immune-related differentially expressed genes (DEGs) were found. Many immune regulatory genes in three classical pathways were found. Antimicrobial peptides, including attacin B, cecropin A, gloverin, lebocin and diapausin, are involved in defending against SM1 challenge, and are mainly produced by Toll and immune deficiency (IMD) pathways. Some melanization genes were changed in *H. cunea*, which suggested that *H. cunea* melanization was activated by SM1. Furthermore, phagocytosis, autophagolysosome and apoptosis pathways in cellular immunity were activated in *H. cunea* against SM1. Finally, the expression patterns of 10 immune genes were analyzed systematically by qRT-PCR, and most of the genes were upregulated compared to the control. Our studies provide useful information about the immune response of *H. cunea* under the stress of SM1, which is important to understand how SM1 affects the immune system of *H. cunea* and provides new ideas to control *H. cunea* by using SM1.

## 1. Introduction

The fall webworm, *Hyphantria cunea* (Drury) (Lepidoptera: Noctuidae) is an important quarantine pest worldwide [1,2]. *H. cunea* spread to Asia in 1945, and was first reported in 1979 in Dandong, Liaoning, China. It is mainly distributed in eastern and northeastern provinces of China, such as Shandong, Henan, Anhui and Jiangsu [3,4]. *H. cunea* is a polyphagous pest that harms nearly 300 kinds of plants, poses a serious threat to ecology and restricts the development of the Chinese agriculture and forestry economy [5,6]. Scientists use a variety of control strategies to alleviate the harm of *H. cunea*, including the use of microorganisms, chemical pesticides, and natural enemies [6]. Microbial insecticides are environmentally friendly and harmless pesticides. Due to their high efficiency in terms of insect control, they have been widely used in global agriculture and forestry.

*Serratia marcescens* Bizio (Enterobacteriaceae: Serratia), is an anaerobic, rod-shaped, short, Gram-negative bacterium that can produce prodigiosin. *S. marcescens*, as a biological control bacterium, can control a variety of agronomically important pests and pathogenic fungi [7,8,9,10]. For example, *S. marcescens* is highly pathogenic and has the ability to control the cotton bollworm, *Helicoverpa armigera* (Hübner) [11]. In previous research, *S. marcescens*, as an extremely effective pathogen, demonstrated pathogenicity in *Spodoptera litura* (Fabricius) [10]. After *S. marcescens* was injected into silkworms, the hemolymph of the silkworm continuously flowed out [12]. *S. marcescens* increases virulence to insects by interacting with the antibacterial protein of the salivary gland in *Riptortus pedestris* (Fabricius) [13]. *S. marcescens* can enhance the control of brown planthoppers in combination with chemical pesticides [14]. The mixture of *S. marcescens* (SM1) and *Metarhizium anisopliae* can increase the lethality of *Odontotermes formosanus* (Shiraki) [15]. In our lab, we found that SM1 exerts insecticidal activity against *H. cunea*, and most of the insects turned red and died after SM1 infection [16]. Unfortunately, the innate immunity of insects could greatly reduce the control effect of microbial insecticides.

Most insects depend on the innate immune system to effectively counter the challenge of pathogens, which greatly reduces their pathogenicity. The insect innate immune system consists of humoral and cellular responses [17]. The humoral immune response includes the synthesis of antimicrobial peptides (AMPs) and the activation of the prophenoloxidase (proPO, PPO) system. The activation of humoral immunity is dependent on the host innate immune receptor pattern recognition receptors (PRRs) [18]. When pathogens infect insects, microbe-associated molecular patterns (MAMPs) bind to PRRs to activate humoral immunity, such as Toll, immune deficiency (IMD) and JAK/STAT, which are conserved across various insect species, indicating that they play an important role in insects [17,19,20,21]. The activation of these pathways is mediated by the host PRRs, such as peptidoglycan recognition proteins (PGRPs), β-1,3-glucan recognition protein (βGRP), lipopolysaccharide (LPS)-binding protein and C-type lectins (CTLs), which can identify the pathogen associated molecular patterns (PAMPs) on the surface of invading microorganisms [22,23,24,25]. Specifically, Gram-negative bacteria and several Gram-positive bacteria containing meso-diaminopimelic acid-type peptidoglycan (DAP-type PGN) activate the IMD pathway, while the Toll pathway is triggered by Gram-positive bacteria, yeasts, and fungi [26,27]. In insects, The JAK/STAT signaling pathways mediate diverse immune responses to microbes, including antibacterial and antiviral [17]. When invading pathogens are recognized by insects, the extracellular serine protease cascade is activated, ultimately activating PPO activating proteases (PAPs) [28]. PAPs can activate inactive PPO by converting it to active phenoloxidase (PO), which can drive the production of melanin to kill pathogens [29]. Therefore, the humoral response plays an important role in insect immune defense systems.

The cellular immune response refers to phagocytosis, autophagy, and apoptosis, which are mediated by circulating hemocytes [17]. Phagocytosis is an evolutionarily conserved cellular immune process that is used by both vertebrate and invertebrate animals for the destruction of small foreign organisms [30]. In both vertebrate and invertebrate animals, autophagy is employed for eliminating the pathogens in the body, including bacteria and viruses [31,32,33]. Apoptosis is a form of programmed cell death. Numerous studies show that apoptosis could protect the insects against the infection of microbe [34,35,36]. Some immune factors, including IMD and inhibitors of apoptosis protein affect the IMD and apoptosis pathways [37].

At present, much work is devoted to searching for effective biocontrol agents for controlling *H. cunea* [5,6]. Our laboratory found that three important detoxifying enzymes (uridine diphosphate-glycosyltransferases, glutathione S-transferases and cytochrome P450s) responded to SM1 by transcriptome sequencing in *H. cunea* [16]. However, the mechanism by which the immune-related genes of *H. cunea* resist SM1 is little understood. Therefore, it is urgent to clarify the interaction mechanism between *H. cunea* and microorganisms. In our study, we attempted to reveal the immune regulation mode of *H. cunea* based on the results of transcriptome sequencing infected by SM1. Three major perspectives (antimicrobial peptide synthesis pathways, melanization and cellular immunity) of immune pathways in *H. cunea* are described in this paper. Our research aims to improve the SM1 control effect and provide theoretical support for its application. We analyzed the interaction between *H. cunea* and SM1 to gain a better understanding of the possible mechanism of *H. cunea* immune response. Thus, this work is an important theoretical basis for the development of new immunosuppressive agents that control *H. cunea*.

## 2. Materials and Methods

### 2.1. RNA Extraction, Synthesis of cDNA Library and Sequencing

Total RNA was extracted from third instar *H. cunea* larvae using the RNAprep Pure Tissue Kit (TIANGEN, Beijing, China) according to the manufacturer’s instructions. The quality and concentration of total RNA were determined using a NanoDrop spectrophotometer (Thermo Fisher Scientific, Waltham, MA, USA). mRNA was isolated from total RNA using poly-T magnetic beads. The first cDNA strand was synthesized utilizing random hexamers, and then second strand cDNA synthesis was performed using dNTPs, DNA Polymerase I and RNase H. The cDNA library was sequenced on an Illumina HiSeqTM 2000 platform.

### 2.2. Sequence Assembly and Annotation

Transcriptome assembly was performed based on high-quality clean data using Trinity (https://github.com/trinityrnaseq/trinityrnaseq/, accessed on 1 September 2021) to produce complete transcripts, and then the longest unigenes were obtained by Tgicl (http://www.tigr.org/tdb/tgi/, accessed on 1 September 2021). The unigenes were aligned with protein databases, such as NR, Swiss-Prot, KEGG and COG. Gene functions were annotated by the BLAST method.

### 2.3. Rearing of S. marcescens

SM1 was stored at −80 °C in an ultra-low temperature refrigerator in our laboratory. SM1 was cultured on solid basal medium in the dark at 30 °C for 12 h. One SM1 colony was picked and placed in 50 mL of seed culture medium, and the culture conditions were 30 °C for 12 h at 200 r/min. Then, the right amount of the seed solution was placed in 200 mL of the fermentation medium, and the shaker was set at 200 r/min and 30 °C for 36 h. The fermentation solution was used for the following experiments. 

### 2.4. Rearing and Treatment of H. cunea

*H. cunea* larvae were collected from poplar planting areas in Huai’an, Jiangsu Province, China. The larvae were reared in a transparent plastic box (20 cm × 14 cm × 10 cm) with fresh poplar leaves at 26 ± 1 °C and a photoperiod of 16 h light: 8 h dark. *H. cunea* was cultivated in the laboratory for 2 generations, then the third instar larvae with similar status were selected as experimental insects, and fresh poplar leaves were soaked in SM1 fermentation solution (treatment) and sterile fermentation medium (control). Every experiment was performed three times. The living larvae of test insects were collected at 20 h, 40 h, 60 h and 70 h respectively and stored at −80 °C. The *H. cunea* larvae fed for 70 h and the control group were used for transcriptome sequencing and verifying the transcriptome data by qRT-PCR. *H. cunea* larvae fed for 20–60 h were used for qRT-PCR.

### 2.5. Quantitative Real-Time PCR (qRT-PCR)

Total RNA was extracted from 50 mg third instar *H. cunea* larvae using TRIzol reagent (Invitrogen, Thermo Fisher Scientific, Waltham, MA, USA) according to the instructions. First strand complementary DNA (cDNA) from 1000 ng of total RNA was synthesized with the PrimeScript RT reagent kit for qRT-PCR (+gDNA remover) (Takara, Dalian, China) following the manufacturer’s protocol. Gene-specific primers were designed by Premier 5.0 software based on the gene sequence (Supplementary Materials Table S1). To judge whether the primers were qualified, LinReg PCR (Version: September 2014) software was used to analyze the qRT-PCR results to determine the actual amplification efficiency of each pair of primers. Ribosomal protein S16 (RPS16) was used as an internal reference. In brief, qRT-PCR was performed in a 20 µL reaction volume containing 1 µL of template cDNA and 10 µL of SYBR Premix Ex Taq kit (TliRNaseH Plus) (Takara, Dalian, China), 0.4 µL of 10 µM forward and reverse primers, 0.4 µL of ROX Reference Dye II and 7.8 µL of ddH_2_O on ABI ViATM 7 Real-time PCR system (Applied Biosystems, FosterCity, CA, USA). The thermal cycling conditions were 95 °C for 30 s, 40 cycles of 95 °C for 5 s, 60 °C for 30 s, and then the melting curve was analyzed. All experiments were independently conducted three times. The relative expression level of *H. cunea* larvae mRNA was calculated by using the 2^−ΔΔC^^t^ method [38,39].

### 2.6. Statistical Analysis

The collected data were analyzed using InStat software (Version 3.05) (GraphPad, San Diego, CA, USA). The statistically significant differences at the *p* < 0.05 (*) and *p* < 0.01 (**) levels were indicated according to one-way analysis of variance (ANOVA). A Student’s *t*-test followed by a two-tailed unpaired *t*-test was used to compare the significant differences among all two samples. 

## 3. Results

### 3.1. Verification of Differentially Expressed Genes (DEGs) from the Transcriptomes by qRT-PCR

**Figure 1:**
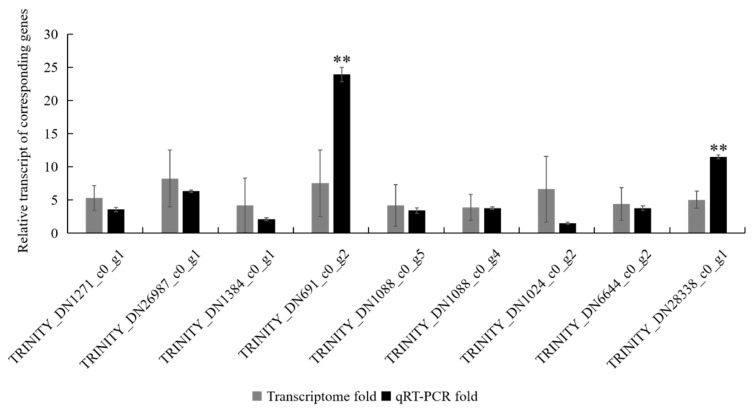
qRT-PCR validation of 9 selected DEGs associated with the immunity of *H. cunea* to confirm the expression pattern detected by sequencing. RPS16 gene was used as an internal reference gene. The qRT-PCR box represents the mean average of specific 2^−ΔΔCt^ values, the transcriptome box represents the log2FoldChange value of each DEG. All genes are shown in the Supplementary Material: Table S2. *p* < 0.01 (**) was considered very significant.

To further evaluate DEGs identified from the transcriptome libraries, nine DEGs were randomly selected from immune-related genes of the *H. cunea* and quantified by qRT-PCR. The RPS16 gene was selected as the internal reference gene for qRT-PCR normalization. The expression of two unigenes (TRINITY_DN691_c0_g2 and TRINITY_DN28338_c0_g1) in qRT-PCR were significantly different from the transcriptome data, but all of the selected unigenes exhibited the same expression trends in qRT-PCR as were observed in the transcriptome data (Figure 1). The results showed that the expression trends presented by qRT-PCR were consistent with the transcriptome library results.

### 3.2. Identification of the Immune-Related DEGs in H. cunea

**Figure 2:**
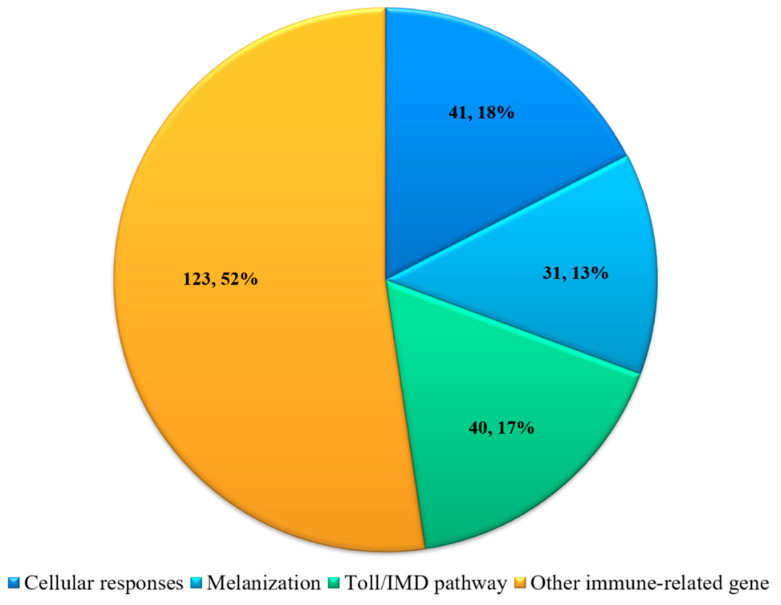
Number and percentage of differentially expressed immune-related genes in *H. cunea* infected by SM1. The distribution number and percent of differentially expressed immune-related genes in each slice are shown on the pie. All genes shown in the Supplementary Material: Table S2.

Immune-related DEGs were identified using combined transcriptome data and qRT-PCR analyses. Initially, parameters were set to identify all immune-related genes present in the transcriptome data. The resulting 234 immune-related differentially expressed transcripts were divided into four main groups (Figure 2): genes related to cellular responses, melanization, immune pathways (AMP synthesis), and other immune-related genes.

For cellular responses, this study identified a total of 41 DEGs in *H. cunea* that could be classified into autophagy, lysosome, apoptosis and cytoskeleton. Twenty-two genes were downregulated and 19 genes were upregulated during SM1 infection (Supplementary Materials Table S2). For melanization, we found 31 DEGs associated with melanization using transcriptome data and qRT-PCR, such as serine proteases and cuticle proteins. For immune pathways (AMP synthesis), 40 genes were identified in *H. cunea* using transcriptome data and qRT-PCR including immune regulators (spatzle, interleukin, etc.) and AMPs (lebocin, attacin, cecropin, gloverin, diapausin). We also identified many other immune-related genes involved in immune responses that changed sharply after *H. cunea* was infected with SM1, such as myrosinase and immune-related gene Hdd. These data indicated that immunity in *H. cunea* was activated after infection with SM1. Based on the above results, we conducted the following experiments to uncover the interaction between *H. cunea* and SM1 at the immune level.

### 3.3. Antimicrobial Peptides Induced by SM1 in H. cunea

**Figure 3:**
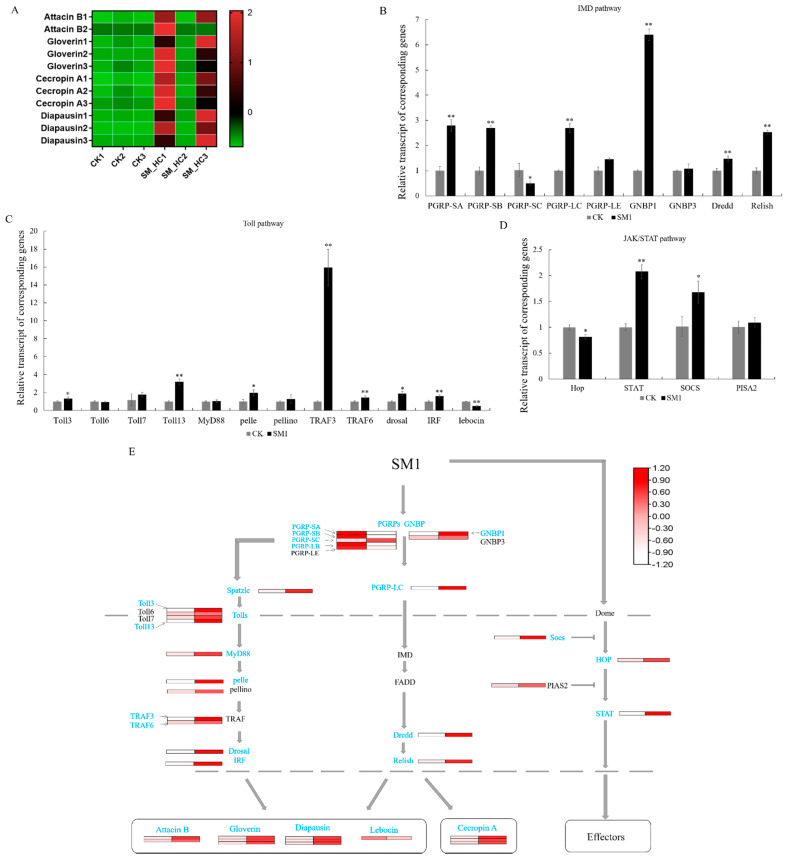
Changes in regulatory patterns of the Toll, IMD and JAK/STAT pathways in *H. cunea* infected by SM1. (**A**) Heatmap clarifying the differences in AMP genes in the Toll/IMD pathway using transcriptome data. CK1, CK2 and CK3: the control groups; SM-HC1, SM-HC2 and SM-HC3: *H. cunea* groups infected with SM1. (**B**) The expression profile of genes in the IMD signal transduction pathway using RT-qPCR. (**C**) The expression profile of genes in the Toll signal transduction pathway using RT-qPCR. (**D**) The expression profile of genes in the JAK/STAT signal transduction pathway using RT-qPCR. (**E**) The immune regulation diagrammatic sketch of the Toll, IMD and JAK/STAT pathways in *H. cunea* integrated the results in (**A**–**D**). Significantly differentially expressed genes are shown in blue. *p* < 0.05 (*) was considered significant, and *p* < 0.01 (**) was considered very significant.

The *H. cunea* immune response system was activated after SM1 infection and the immune signal was amplified to produce antimicrobial peptides (AMPs) or other effectors. Finally, the bacteria were killed. The Toll pathway, IMD pathway, and JAK/STAT pathway are the three most canonical immune pathways in insects. However, for *H. cunea*, little is known about the corresponding pathway and how it works. To clarify the immune regulatory network of *H. cunea*, transcriptome data analysis and qRT-PCR experiments were performed, and the regulation patterns of the three major regulatory pathways were roughly obtained. Five kinds of AMPs were found in our data, including lebocin, attacin B, gloverin, cecropin and diapausin (Figure 3A), as well as DEGs in *H. cunea* infected by SM1, which were the effectors of the Toll/IMD pathway. Most of the AMPs were upregulated, which indicated that the insect immune system blocked SM1 infection. The immune system is controlled by many regulatory factors in the pathway. In IMD, *H. cunea* PRRs were induced by PAMPs, including PGRPs and Gram-negative binding proteins (GNBPs). PGRP-SA/SB/LC and GNBP1 were upregulated, and PGRP-SC was downregulated (Figure 3B). Dredd and Relish (as important regulatory genes in the IMD pathway) were upregulated, which could transfer signals into the nucleus and stimulate the production of AMPs. In the Toll pathway, spatzle was activated by extracellular recognition factors, such as PGRPs and GNBPs (Figure 3E). Therefore, the anti-SM1 process of Toll was initiated, and the toll receptor (Toll 3/13) was upregulated. Then, pelle, TRAF3/6, dorsal and interferon regulatory factor 2-binding protein-like A (IRF) amplified immune signaling and promoted the production of AMPs (Figure 3C,E). In the JAK/STAT pathway, hopscotch (hop) was downregulated, and the signal may be inhibited by SOCS. STAT at the end of the JAK/STAT pathway was upregulated, so we thought that the JAK/STAT pathway was activated (Figure 3D). However, in our data, we could not find any effectors in the JAK/STAT pathway. In total, the three most well characterized immune signaling pathways of *H. cunea* are activated, and AMPs play an essential role in the defense against SM1 infection.

### 3.4. Genes of the Melanization Pathway Induced by SM1 in H. cunea

**Figure 4:**
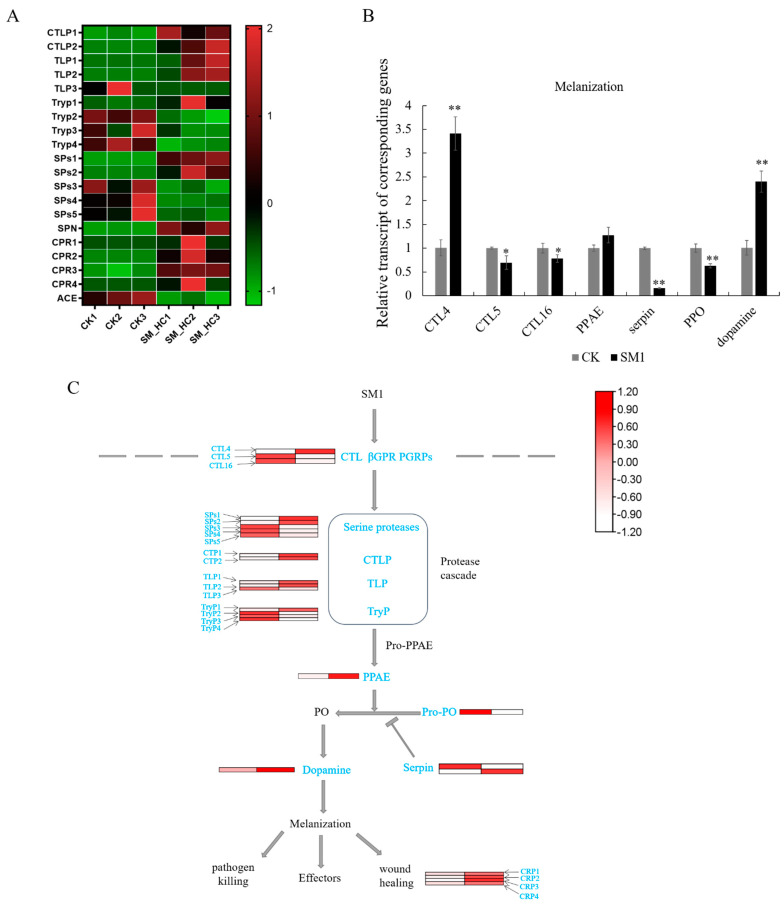
Changes in the regulatory patterns of the melanization in *H. cunea* infected by SM1. (**A**) Heatmap clarifying the differences in biochemical pathway genes in melanization using transcriptome data. CK1, CK2 and CK3: the control groups. SM-HC1, SM-HC2 and SM-HC3: *H. cunea* groups infected with SM1. (**B**) The expression profile of genes in melanization in *H. cunea* infected with SM1 and the control using RT-qPCR. (**C**) Diagrammatic sketch of melanization in *H. cunea* integrated the results in (**A**,**B**). Significantly differentially expressed genes are shown in blue. *p* < 0.05 (*) was considered significant, and *p* < 0.01 (**) was considered very significant.

Melanotic encapsulation (the production and deposition of melanin pigments on the surface of pathogens or parasites) is a common phenomenon found in many arthropods. Arthropod melanization is controlled by a cascade of serine proteases that ultimately activates the PPO, and the PPO activation cascade is negatively regulated by serpins. After SM1 infection, the genes involved in the melanization process changed. Chymotrypsin-like serine protease (CLIP) (Figure 4A,C), as a digestive enzyme in immune cascade pathways, was highly expressed in insects with SM1. We also found that the CLIP precursor was induced to upregulate the expression compared to the control. Serine proteases (SPs) (Figure 4A,C), as important virulence factors for pathogenic microbes, were regulated after SM1 infection. In this study, the expression levels of two of the five SPs, SPs1 and SPs2, were upregulated. These results indicated that the melanization pathway was activated by SM1. Trypsin-like serine protease (TLP), as a regulator in this pathway, could activate the immune response and inhibit bacterial growth. The expression of two trypsin-like serine proteases was upregulated in our data. However, serine protease inhibitor (SPN), a negative regulator in the serine protease cascade pathway, was induced during the defense process in *H. cunea* (Figure 4A). The cuticle is a vital component for the formation of the melanic color pattern. Four cuticular proteins were upregulated after SM1 infection, which showed that the ability to resist pathogens was activated. Then, we performed qRT-PCR detection of the expression levels of regulatory genes in melanization. CTLs, such as CTL4/5/16, were changed significantly. Only CTL4 was upregulated in insects during SM1 infection, while CTL5/16 were downregulated in *H. cunea* (Figure 4A). One PPO gene was downregulated when *H. cunea* was infected by SM1. In addition, dopamine was upregulated by SM1, which was an important regulator of the end regulatory gene in melanization (Figure 4B). Moreover, angiotensin converting enzyme (ACE) acted as a negative regulator of melanization, which was involved in regulating the activity of PO. ACE was down-regulated after SM1 infection in this research (Figure 4A). All in all, these results indicated that melanization in *H. cunea* was activated to defend against SM1 infection.

### 3.5. Induction of the Cellular Immune Response by SM1

**Figure 5:**
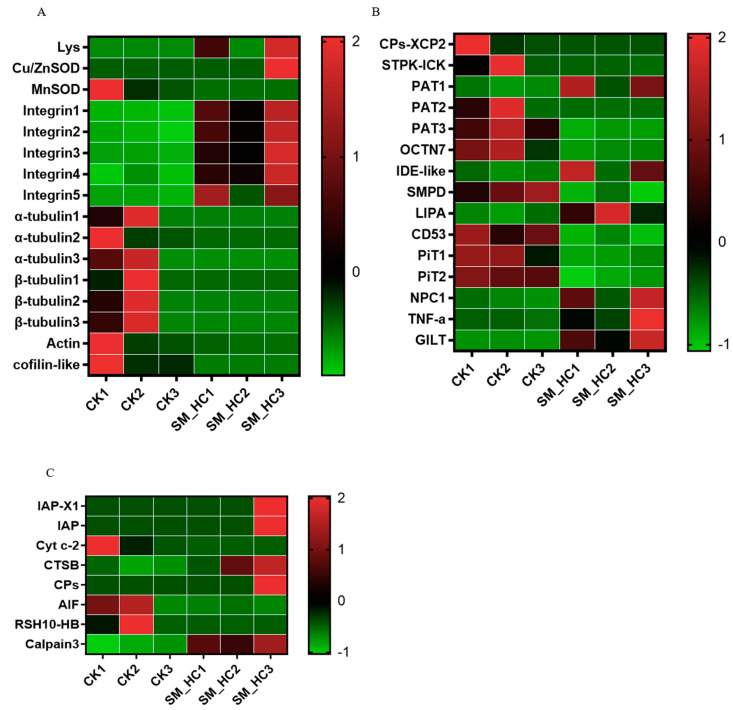
Heatmaps clarifying differences of genes involved in the cellular response using transcriptome data. (**A**) Cluster of cellular phagocytosis families and cytoskeletal genes. (**B**) Cluster of cellular autophagolysosome families. (**C**) Cluster of cellular apoptosis families. CK1, CK2 and CK3: the control groups. SM-HC1, SM-HC2 and SM-HC3: *H. cunea* groups infected with SM1.

Many genes known to be involved in phagocytosis, the formation of autophagolysosomes, apoptosis and some cytoskeletons were also detected (Figure 5). In our results, several phagocytosis genes were identified (Figure 5A). One lysozyme gene was largely upregulated, and five integrin genes were also significantly upregulated compared with the uninfected larvae. The phagocytosis response requires host cell cytoskeletal remodeling. In our study, many DEGs involved in phagocytosis including lysozyme, superoxide dismutase (SOD), integrins and cytoskeletal tubulin, actin, and cofilin, were highly expressed after *H. cunea* infection with SM1. Integrin, as a vital phagocytic component, was upregulated to defend against SM1 infection. In addition, several cytoskeleton genes were downregulated, perhaps due to their participation in *H. cunea* cell phagocytosis to defend against the infection of SM1. These data suggested that the phagocytosis response was stimulated to fight against SM1 bacteria. Some DEG genes in the autophagolysosome pathway were detected in *H. cunea* (Figure 5B), such as LPS-induced tumor necrosis factor-a (TNF-a), cysteine proteases XCP2 (CPs-XCP2), gamma-interferon-inducible lysosomal thiol reductase-like (GILT) and sphingomyelin phosphodiesterase 1-like. CPs-XCP2 was changed during SM1 infection, which suggested that the formation of autophagolysosomes in *H. cunea* inhibited SM1 infection. Apoptosis is an important mechanism in defense against microbial pathogens. Some important DEG genes of apoptosis in *H. cunea* were identified (Figure 5C), including inhibitor of apoptosis proteins (IAPs) and apoptosis-inducing factor (AIF) cysteine protease, and played important roles in resisting the infection of SM1 in *H. cunea*. Our data suggest that apoptosis is induced by SM1 in *H. cunea*. In the *H. cunea* cell response, phagocytosis, autophagolysosomes and apoptosis are effective strategies to prevent SM1 infection.

### 3.6. The Response of Immune-Related Genes to SM1 in H. cunea

**Figure 6:**
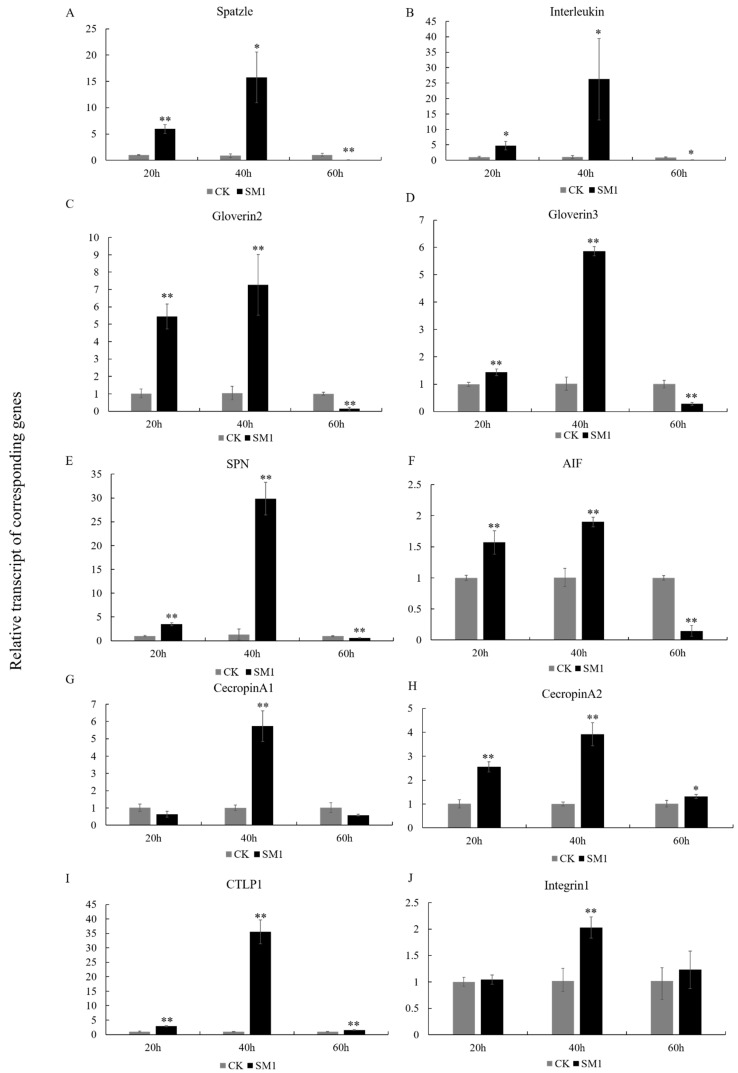
Expression profile of immune-related genes in *H. cunea* infected with SM1. All relative expression levels were transformed by the 2^−ΔΔ^^Ct^ method. Two-tailed unpaired *t*-tests were used to analyze the significant differences between the control (CK) and *H. cunea* infected with *S. marcescens* (SM1), *p* < 0.05 (*) was considered significant, and *p* < 0.01 (**) was considered very significant. (**A**) Spatzle; (**B**) Interleukin; (**C**) Gloverin2; (**D**) Gloverin3; (**E**) Cecropin A1; (**F**) Cecropin A2; (**G**) CTLP1; (**H**) SPN; (**I**) AIF; (**J**) Integrin1.

The expression patterns of 10 immune-related genes (spatzle, Interleukin, gloverin2, gloverin3, cecropin A1, cecropin A2, CTLP1, SPN, AIF, Integrin1) were analyzed at different infected time in *H. cunea* using qRT-PCR (Figure 6).

When *H. cunea* was infected for 20 h, spatzle (t = 10.37, df = 4, *p <* 0.01), Interleukin (t = 4.581, df = 4, *p <* 0.05), gloverin2 (t = 9.91, df = 4, *p <* 0.01), gloverin3 (t = 5.384, df = 4, *p <* 0.01), SPN (t = 12.22, df = 4, *p <* 0.01) and AIF (t = 5.148, df = 4, *p <* 0.01) were induced by SM1 compared with the control groups (Figure 6A–F); when *H. cunea* was infected for 40 h, the expression of spatzle (t = 2.779, df = 4, *p <* 0.05), Interleukin (t = 3.299, df = 4, *p <* 0.05), gloverin2 (t = 6.018, df = 4, *p <* 0.01), gloverin3 (t = 28.42, df = 4, *p <* 0.01), SPN (t = 13.66, df = 4, *p <* 0.01) and AIF (t = 9.461, df = 4, *p <* 0.01) was also upregulated compared with the control; when *H. cunea* was infected for 60 h, the expression of spatzle (t = 6.12, df = 4, *p <* 0.01), Interleukin (t = 6.179, df = 4, *p <* 0.05), gloverin2 (t = 12.56, df = 4, *p <* 0.01), gloverin3 (t = 8.279, df = 4, *p <* 0.01), SPN (t = 5.977, df = 4, *p <* 0.01) and AIF (t = 15.45, df = 4, *p <* 0.01) was downregulated compared with the control. In *H. cunea*, the expression of immune genes was induced by SM1 in the early stage, which is helpful for resisting SM1 infection. With the continuous infection of SM1, the expression of immune genes in *H. cunea* was gradually strengthened. Finally, the accumulation of SM1 and the strengthening of toxic factors destroyed the immune system of *H. cunea*.

A few genes of *H. cunea* also showed different expression patterns after infection with SM1. At 20 h, cecropin A1 (t = 2.374, df = 4, *p* > 0.05) and Integrin1 (t = 0.6066, df = 4, *p* > 0.05) were not different compared with the control (Figure 6G,J); at 40 h, cecropin A1 (t = 9.084, df = 4, *p <* 0.01) and Integrin1 (t = 5.579, df = 4, *p <* 0.01) were induced by SM1 compared with the control. At 60 h, cecropin A1 (t = 2.608, df = 4, *p* > 0.05) and Integrin1 (t = 0.8382, df = 4, *p* > 0.05) were not different compared with the control. Cecropin A2 (t = 9.781, df = 4, *p <* 0.01) and CTLP1 (t = 12.94, df = 4, *p <* 0.01) were upregulated compared with the control at 20 h (Figure 6H,I); cecropin A2 (t = 10.34, df = 4, *p <* 0.01) and CTLP1 (t = 14.46, df = 4, *p <* 0.01) were upregulated compared with the control at 40 h; cecropin A2 (t = 3.358, df = 4, *p <* 0.05) and CTLP1 (t = 6.323, df = 4, *p <* 0.01) were also upregulated compared with the control at 60 h. In total, the results showed that 10 immune genes were upregulated in *H. cunea* during SM1 infection, and these genes play an important role in the process of resisting SM1.

## 4. Discussion

*H. cunea* is one of the most destructive agricultural and forestry pests in the world. The harm caused by chemical pesticides to the environment has attracted increasing attention from scientists studying microbial pesticides. Exploiting microbes (such as bacteria, fungi and viruses) to kill insects is a promising strategy for controlling pests [40,41]. Here, we found that SM1 could kill *H. cunea* larvae; however, the interaction between insects and SM1 is poorly understood. In this study, we combined transcriptome data and related experimental results to analyze how *H. cunea* responds to SM1 infection. Changes in the *H. cunea* larvae during SM1 infection directly reflect the impact of the bacteria on the host responses, and the theory in this field could be refined to develop biological control agents.

### 4.1. AMPs Synthesis Pathway Response to SM1 in H. cunea

*H. cunea* and pathogen interactions have been reported in bacteria–insect and virus–insect systems [6,42], and the *H. cunea* detoxifying enzyme system responses to SM1 were reported [16]. However, the immune mechanism of insect hosts interacting with pathogens is not well understood. The immune pathways of Toll, IMD and JAK/STAT are three classical defense modes of insects, and the three immune pathways in *H. cunea* were activated by SM1. At present, we have clarified the immune regulation mechanism of *H. cunea* to reveal the interaction between *H. cunea* and SM1.

The activation of the immune pathway relies on a series of PRRs to recognize PAMPs and then induce appropriate effector responses to remove the infection [43]. In the IMD pathway, PGRP-LC is a transmembrane receptor that preferably binds DAP-type PGN on Gram-negative bacteria, and PGRP-LC interacts with IMD to enhance the immune signal [44,45]. In our study, PGRP-LC was largely upregulated in *H. cunea* (Figure 3E). Dredd, as a vital regulator in the IMD pathway, was activated by the ubiquitination of the E3-ligase inhibitor of apoptosis 2 [46]. Dredd cleaved IMD and Relish, and then the signal was delivered to the nucleus. In our study, Dredd and Relish were upregulated in *H. cunea*. Finally, *H. cunea* released AMPs to protect against SM1 infection (Figure 3B,E). These results suggested that the IMD pathway was activated in *H. cunea* after infection with SM1. For the Toll pathway, PGRP-SA and PGRP-SD were induced in this pathway (PGRP-SA will bind to GNBP1), which could activate the Toll pathway. Spatzle binds the Toll receptor [47], and then the signal transfers to the core components of the Toll pathway (Myd88, pelle, pellino, dorsal, etc.) [48]. Finally, the Toll signal enters the nucleus and produces AMPs. In our research, most of the genes in the Toll pathway of *H. cunea* were differentially expressed (Figure 3C,E). For example, GNBP1 was upregulated in *H. cunea* during SM1 infection (Figure 3E). Gram-negative bacteria can induce the expression of silkworm GNBP [49]. Therefore, we believe that the GNBP of *H. cunea* could be induced by SM1 and transmit the signal downstream of the Toll pathway. Most reports indicate that the Toll pathway is activated by fungi and Gram-positive bacteria [26,47]. Our results indicated that SM1 could also activate the Toll pathway. In our study, we obtained five kinds of AMPs from our transcriptome, including cecropin A, gloverin, attacin B, diapausin and lebocin. Cecropin A, only in the IMD pathway, also interact with negatively charged bacterial cell membranes [50]. Cecropin A was upregulated in *H. cunea* after SM1 infection (Figure 3A). In previous study, lebocin was also induced by Gram-negative bacteria in silkworms [51], but the expression of lebocin was downregulated in this study (Figure 3A). We speculated that the lebocin gene of *H. cunea* does not play a major role in defense against SM1at 70 h. Gloverins show a broad spectrum of antimicrobial activity, with some members of this family only being active against Gram-positive bacteria and others only against Gram-negative bacteria or viruses [51]. When SM1 infects *H. cunea*, the expression of gloverins could be induced to be upregulated by the bacteria, which indicates that gloverins have the ability to kill SM1 (Figure 3A,E). *H. cunea* attacin B was active against Gram-negative bacteria such as *E. coli* and *Citrobacter freundii*, as well as the fungus *C. albicans* [52]. In our study, attacin B was upregulated in *H. cunea* after SM1 infection (Figure 3A,E), which is consistent with previous studies [52]. Diapausin-1 exhibits antifungal activity in *Manduca sexta* [53]. Diapausin was also upregulated in *H. cunea* (Figure 3A,E), which shows the activity of anti-SM1. However, the antibacterial mechanism of AMPs in *H. cunea* needs further study. Similar to the Toll pathway and IMD pathway, the JAK/STAT pathway is involved in both immunity and development [17]. The hop, STAT and SOCS genes of JAK/STAT pathway were induced by SM1 in this study (Figure 3D,E). However, we could not find any effectors in this pathway. Therefore, we speculate that this immune pathway of *H. cunea* may be weakly induced by SM1.

Numerous previous studies suggest that Gram-negative bacteria and several Gram-positive bacteria activate the IMD pathway, while the Toll pathway is initiated by Gram-positive bacteria, yeasts, and fungi [26,27]. *Bacillus bombysepticus* infections can induce a weak JAK/STAT pathway response in silkworm [54]. By analyzing the expression of immune regulatory genes in the Toll, IMD and JAK/STAT pathways, we found that all three pathways of *H. cunea* were activated. However, when we analyzed the expression of the end effector products of the three immune pathways, we determined that the Toll and IMD pathways play a major role in the protection of *H. cunea* against SM1 infection, while the JAK/STAT pathway plays a small role in this process.

### 4.2. SM1 Infection Induced the Melanization in H. cunea

ProPO cascade melanization is an effective innate immune system, and plays an important role in wound healing, killing microorganisms and facilitating melanotic encapsulation of parasites [55]. Serine protease and serine protease inhibitors regulate diverse immune mechanisms including proPO cascade melanization. For example, in *Ostrinia furnacalis* and *M. sexta*, serine protease inhibitors mediate the protease cascades of the proPO activation cascade and Toll signaling pathway [56,57]. Here, we found that the genes associated with the *H. cunea* proPO cascade melanization pathway were upregulated, such as serine protease, serine protease inhibitor, chymotrypsin-like serine protease and trypsin-like proteinase T2b precursor (Figure 4C). These results were consistent with observations in *Bombyx mori* infected by *B. bombysepticus* and indicated that the proPO cascade melanization pathway was significantly activated after infection [54]. In addition, several other key enzymes during the silkworm melanization process, such as trypsin-like serine protease and trypsin-like protein also were modulated. In a previous study, ACE (a negative regulator of melanization) was found to fine-tune the immune response by inhibiting the activity of PO in locusts migratoria [58]. ACE was downregulated in *H. cunea* after infected with SM1 in this study (Figure 4A). In total, our results implied that proPO cascade melanization rapidly mediates immune defense responses upon microbial infection in *H. cunea*.

### 4.3. SM1 Infection Affects Cell Responses in H. cunea

After SM1 entered the *H. cunea* hemolymph, the cellular immune response was triggered (Figure 5). The cellular response refers to phagocytosis, encapsulation, apoptosis, autophagolysosome and nodule formation, which are mediated by hemocytes [59]. The phagocytosis response required host hemocyte cytoskeletal remodeling (Figure 5A). In this study, we found that many DEGs involved in superoxide dismutase, integrin, lysozyme and cytoskeleton reorganization including alpha-tubulin, actin and cofilin, were significantly regulated. Nagaosa and colleagues showed the implication of βv-integrin in the phagocytosis of both apoptotic cells and *S. aureus* in Drosophila [60]. The integrins of *H. cunea* were induced by SM1, which showed that phagocytosis was started (Figure 5A). In other research, the phagocytic activity of hemocytes of both *G. mellonella* and *B. mori* larvae was also significantly enhanced after immune priming with bacteria [61,62]. Combining the abovementioned results, we think that the hemocyte phagocytosis of *H. cunea* was activated to fight against the invading bacteria.

Apoptosis is a genetically and biochemically controlled process of cell death and plays vital roles in the development, tissue homeostasis and defense of multicellular organisms by selectively removing unwanted or damaged cells [63,64]. Apoptosis is often induced by microorganisms in the early stage to minimize their replication within host cells. Mitochondria play a key role in the regulation of apoptosis (cell death) [65,66]. In our study, inhibitors of apoptosis proteins, apoptosis-inducing Factor 1, cysteine protease and cathepsin B involved in apoptosis, were modulated after SM1 infection (Figure 5B). In summary, we believed that the apoptosis of *H. cunea* is activated by SM1.

When an organism is infected by microorganisms, the autophagosome is formed and then fused with lysosomes to clear them [67]. Here, we found that genes associated with the *H. cunea* autophagolysosome pathway were changed (Figure 5C), such as cysteine protease XCP2, serine/threonine-protein kinase, insulin-degrading enzyme-like, etc. These data indicated that autophagolysosomes rapidly mediate immune defense responses during SM1 infection.

## 5. Conclusions

In the current research, the possible immune-regulated mechanism between *H. cunea* and SM1 were analyzed from humoral immunity and cellular immunity using transcriptome sequencing and qRT-PCR. In humoral immunity, the Toll pathway and IMD pathway work together to resist SM1 infection; the melanization pathway also plays an important role in the anti-SM1 process. In cellular immunity, phagocytosis pathways, autophagolysosome pathways and apoptosis pathways were induced in *H. cunea* against SM1 infection. This study uncovers the immune mechanism of *H. cunea* against SM1 and establishes theoretical support for improving the control effect of SM1 and its application. Furthermore, it provides a basis for the development of more effective biological control technology for *H. cunea*.

## Data Availability

The data presented in this study are available on request from the corresponding author.

## Supplementary Materials

The following are available online at https://www.mdpi.com/article/10.3390/insects12110983/s1, Table S1: Primers used in qRT-PCR, Table S2: Differentially expressed immune-related genes in *H. cunea*.
